# Fitness consequences of female multiple mating: A direct test of indirect benefits

**DOI:** 10.1186/1471-2148-12-185

**Published:** 2012-09-15

**Authors:** Miguel Barbosa, Sean R Connolly, Mizue Hisano, Maria Dornelas, Anne E Magurran

**Affiliations:** 1 CESAM, Department of Biology, Universidade de Aveiro, Campus de Santiago, Aveiro, 3810, Portugal; 2 ARC Centre of Excellence for Coral Reef Studies, James Cook University, Townsville, 4811, QLD, Australia; 3 Scottish Oceans Institute, School of Biology, University of St Andrews, East Sands, St Andrews, KY16 8LB, Fife, United Kingdom

**Keywords:** Selection, Fitness, Benefits, Sex ratio, Growth rate, Size at birth, Mate choice, Multiple mating

## Abstract

**Background:**

The observation that females mate multiply when males provide nothing but sperm - which sexual selection theory suggests is unlikely to be limiting - continues to puzzle evolutionary biologists. Here we test the hypothesis that multiple mating is prevalent under such circumstances because it enhances female fitness. We do this by allowing female Trinidadian guppies to mate with either a single male or with multiple males, and then tracking the consequences of these matings across two generations.

**Results:**

Overall, multiply mated females produced 67% more F2 grand-offspring than singly mated females. These offspring, however, did not grow or mature faster, nor were they larger at birth, than F2 grand-offspring of singly mated females. Our results, however, show that multiple mating yields benefits to females in the form of an increase in the production of F1. The higher fecundity among multiply mated mothers was driven by greater production of sons but not daughters. However, contrary to expectation, individually, the offspring of multiply mated females do not grow at different rates than offspring of singly mated females, nor do any indirect fitness benefits or costs accrue to second-generation offspring.

**Conclusions:**

The study provides strong evidence that multiple mating is advantageous to females, even when males contribute only sperm. This benefit is achieved through an increase in fecundity in the first generation, rather than through other fitness correlates such as size at birth, growth rate, time to sexual maturation and survival. Considered alongside previous work that female guppies can choose to mate with multiple partners, our results provide compelling evidence that direct fitness benefits underpin these mating decisions.

## Background

Female multiple mating is prevalent in nature, even when males provide no material benefits such as food or parental care to females [1-3]. Female multiple mating in such circumstances is unexpected because mating carries associated costs. Aside from the energy and time required to engage in mating [4], multiply mated females may significantly increase their risk of predation or disease transmission [5]. Understanding why female multiple mating is the rule rather than the exception in the absence of material benefits remains a key challenge in evolutionary ecology [6,7].

The adaptive significance of multiple mating has been extensively debated and the general idea is that, to be adaptive, the costs of multiple mating must be offset by benefits that enhance female fitness. Two types of benefits are commonly used to explain the adaptive value of multiple mating: non-genetic benefits (direct/first generation) [1] and genetic benefits (indirect/second generation) [8]. Direct benefits derive from the quality of the sperm of certain males that may increase female fecundity, longevity, or mating rate [1]. Additionally, if males transfer insufficient sperm, females may mate multiply to ensure all eggs are fertilized, hence obtaining fecundity benefits [9]. Indirect benefits, on the other hand, are next generation benefits that are associated with post copulatory sexual selection mechanisms that are promoted by mating with multiple, genetically variable, males (i.e., sperm competition and cryptic choice) [8,10,11]. Post copulatory sexual selection can select for compatible genes [12], thus reducing inbreeding depression [13] and leading to the production of offspring of higher quality [6]. Alternatively, post mating sexual selection may also favour males with competitive ejaculates to sire more competitive offspring [14].

Although, direct benefits play a crucial role in the adaptiveness of female multiple mating [11], over the last decade there has been an increasing amount of theoretical and empirical evidence in support of the adaptive value of multiple mating based on indirect benefits [8]. Examples of indirect benefits include, increased offspring attractiveness and viability [6,15,16], genetic heterogeneity and phenotypic diversity [17,18]. Indirect benefits have been proposed to be sufficient to maintain multiple mating even at the expense of direct costs to females, such as reduced longevity [19], if the immediate direct costs are outweighed by a sufficiently large increase in indirect fitness benefits, (i.e., second-generation benefits).

Despite evidence that females gain indirect benefits (i.e., second-generation benefits) from multiple mating, two difficulties are often identified. First, it is difficult to disentangle direct and indirect benefits. The mechanism by which females obtain direct benefits, such as increased fecundity, may also affect offspring viability, thereby obscuring any evidence of indirect benefits [20,21]. Second, indirect benefits assume that F1 offspring fitness is elevated [22]. However, to date, most tests of indirect benefits have focused on offspring traits in the first generation (F1), rather than on the relationship between multiple mating and an increase in the numerical representation in future generations (net fitness) (but see, [6,23,24], for examples of studies using net fitness). Such demonstration is vital to confirm the underlying assumption that indirect benefits result in an increase in F1 offspring fitness [22].

In order to circumvent these difficulties, a stronger test of the adaptiveness of multiple mating would be one that: 1) tracks the fate of offspring across two generations [25,26], and 2) teases apart first-generation and second-generation fitness benefits. The number of grand-offspring reaching reproductive maturity is a robust measure of fitness [27,28]. To partition the first and second generation contributions by this predicted benefit of multiple mating, we need to know the extent to which the production of grand-offspring is attributable to an increase in the numbers of offspring produced and the extent to which it results from improved offspring fitness (i.e., an indirect effect).

Quantifying the benefits of multiple mating solely on the basis of the number of offspring produced, however, may produce biased estimates of fitness. Fitness is a function of the number of viable descendants produced, as well as the influence that other life history traits have on the performance of offspring in particular contexts, or at a given point in the life cycle [28,29]. If, for example, offspring from multiply mated females are larger at birth or grow faster (two fitness correlates) than offspring from singly mated mothers, then multiply mated females could attain greater fitness benefits for the same number of offspring produced. Consequently, fitness should be complemented with information on survival and how this is affected by other life history traits known *a priori* to be correlated with fitness [30].

Here, we test the hypothesis that multiple mating results in increased F1 fitness, by examining the number of ‘grand-offspring’ produced. We develop and apply a multi-generational test that allows us to disentangle the contributions of first generation (direct) and second-generation (indirect) effects of this outcome. Specifically, using the Trinidadian guppy (*Poecilia reticulata*), we conduct a two-generation experiment to assess the net fitness of sons and daughters produced by contrasting mating (single vs. multiple) treatments. Guppies have a promiscuous non-resource-based mating system, in which female multiple mating is extremely common [31,32], with the highest total number of putative sires per brood recorded for a vertebrate species [33]. While male sexual harassment is important in the cost-benefit trade-off of female mating decisions [2,34], numerous studies have shown that, under some circumstances, females promote multiple matings [35-37]. This suggests that multiple mating cannot be exclusively attributed to a “convenience strategy” where females approach males to minimize costs associated with sexual harassment from other males [38]. Two other conditions may justify the adaptive value of multiple mating in the absence of material benefits: increasing female fecundity (direct benefits) and/or increasing offspring reproductive success (indirect benefits) [39]. Given the ubiquity of female multiple mating in guppies, and coupled with the absence of any resource-based/material benefits from males to females, we predict that under identical social and environmental conditions, multiply mated females will produce more grand-offspring than singly mated ones.

We first tested the prediction that multiple mated females obtain indirect benefits by producing more grand-offspring (F2). We did this by comparing the number of F2s generated via single and multiple mated F0 treatments. We then examined how mating success and brood size of F0 females and F1 offspring contributed to this effect. First, the number of F1s produced was compared between the two mating treatments. Second, we tested whether the F1 offspring of multiply mated females were more viable than those of singly-mated females, when all F1s were paired with randomly selected mates under a common garden experimental design. This allowed us to attribute any overall differences in the number of grand-offspring produced to; having more F1 offspring, to having F1 offspring that were more viable, or to a combination of both. In addition, to assess the extent to which these fitness measures may be biased by differences in offspring characteristics, we measured size at birth and growth rates, which have been previously described as important fitness correlates. Differences between the offspring of single and multiple mated females in these quantities could, in nature, offset differences in the number of F1 or F2 offspring produced.

## Results

### F0 to F2

**Figure 1:**
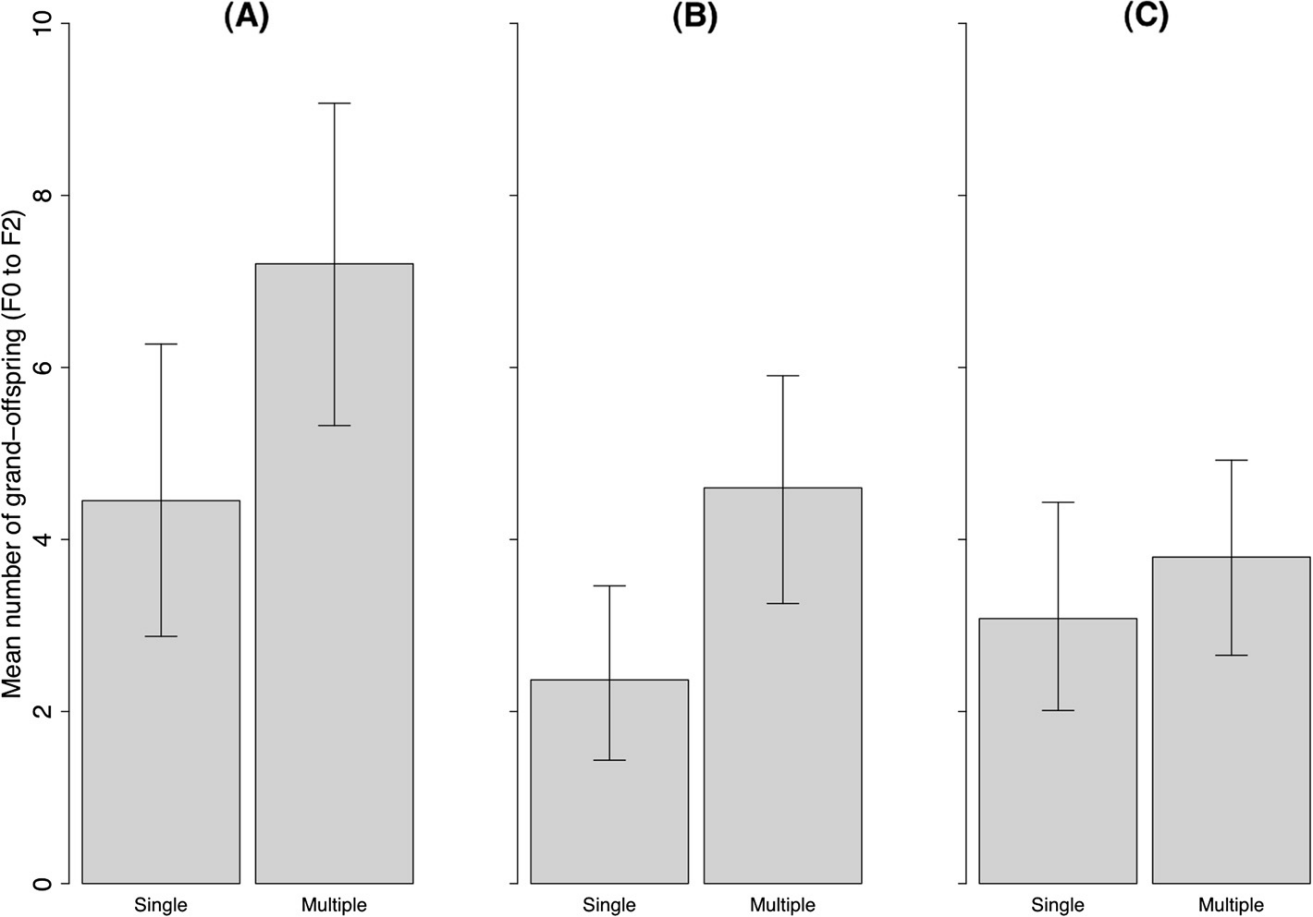
**Indirect Fitness (F0 to F2).** Mean number of viable grand-offspring (F2) produced by a singly or multiply mated F0 females via **(A)** all F1, **(B)** male F1, and **(C)** female F1. Values in **(B)** and **(C)** do not sum to the values in **(A)**, because not all F0s produced mixed sex broods. Whiskers indicate 95% bootstrap percentile confidence intervals. Sample sizes used to calculate the means are shown in Supporting Information 1.

The estimated probability of breeding success for F0 females (i.e., of successfully producing a first brood) was higher for multiple (0.67) than for single (0.55) matings, but the difference was not statistically significant (likelihood ratio test: *R =* 2.1, *P =* 0.15) (Table 1). However, once the number of viable grand-offspring produced (i.e., number of F2 that survived until sexual maturation) was taken into account, multiply mated F0 females produced, on average, 67% more viable grand-offspring (F2) than singly mated F0 females (Figure 1A; *P <* 0.05). Separate analysis of grand-offspring produced via female and viable male F1 individuals indicated that this was principally due to the fact that multiple matings produced more than twice as many grand-offspring as single matings, on average, via viable male F1 (Figure 1B), a highly significant difference (*P <* 0.01). In contrast, the grand-offspring produced via female F1 did not differ significantly with mating treatment (Figure 1C; *P =* 0.16). Note that the mean number of F2s descended via a singly or multiply mated grandmother through a son or daughter F1 was calculated using only viable F1s.

**Table 1:** Effect of Mating Treatment on Components of Fitness

**Fitness Variable**	**Mating treatment**	**N**			**Mean**			**SEM**		
		**F0**	**F1**	**F2**	**F0**	**F1**	**F2**	**F0**	**F1**	**F2**
Brood Size	Single	40	121	335		3.02	2.83	0.28	0.20	
	Multiple	39	154	436		4.00	3.00	0.36	0.16	
Breeding Success	Single	73(.55)	121(.70)		32.7	82.2		0.55	0.46	
	Multiple	58(.69)	154(.75)		18.0	116		0.43	0.40	
Growth Rate	Single		121	144		0.12	0.12		.003	.002
	Multiple		148	160		0.11	0.13		.002	.003
Size at Birth	Single		121	144		0.85	0.86		0.06	0.07
	Multiple		147	148		0.86	0.86		0.06	.004
Sexual Maturation	Single		58	158		45.6	40		1.65	0.77
	Multiple		91	187		47.8	42		1.43	0.76
Mortality	Single	3	8	15						
	Multiple	2	10	14						

Sample size (N), Mean and Standard Error of the Mean for single and multiple mating treatments at F0, F1 and F2, for each component of fitness. Mean brood size for F2 was calculated using only viable F1s (i.e., individuals surviving to 12 weeks). Breeding success in brackets is the fitted probability of producing a brood, based on a negative binomial distribution for F0 and a binomial probability distribution for F1. The means and standard error of means for breeding success were calculated using these two probability distributions. For all other variables arithmetic means are presented. Growth rate was calculated as the rate of weekly growth (cm) over 12 weeks. Maturation is the number of days from birth until sexual maturation. Mortality is the number of individuals that died per mating treatment before producing a first brood.

### F0 to F1

**Figure 2:**
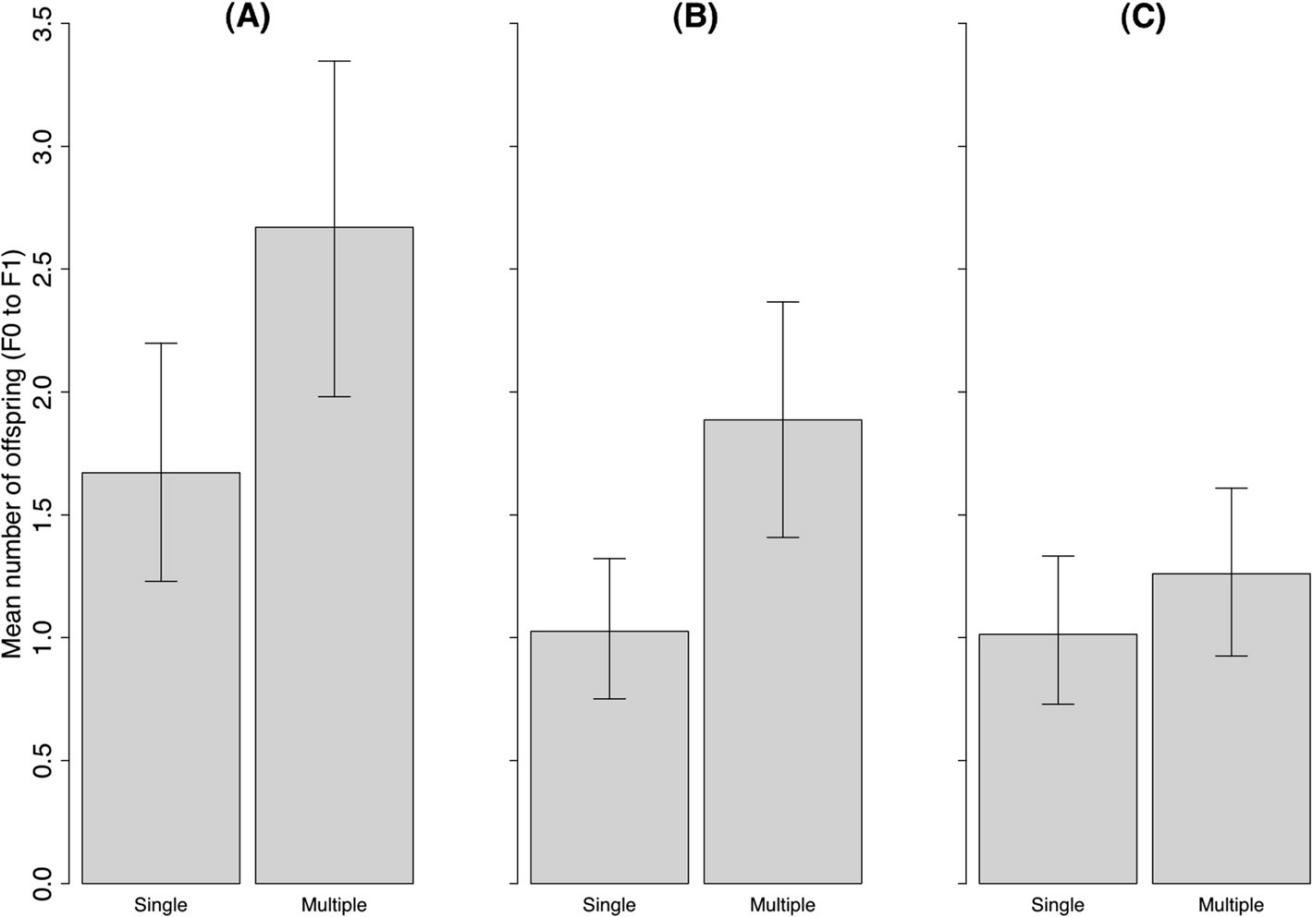
**Direct Fitness (F0 to F1).** Mean number of viable offspring (F1) produced by singly or multiply mated F0 females, counting **(A)** all F1, **(B)** male F1, and **(C)** female F1. Means were calculated using only those F1s that reached sexual maturity. Whiskers indicate 95% bootstrap confidence intervals. Sample sizes used to calculate the means are shown in Supporting Information 1.

Multiply mated females produced 60% more viable F1 offspring, on average, than singly mated females (Figure 2A; *P <* 0.01). The greater fecundity observed was driven by the production of 83% more viable males (Figure 2B: *P <* 0.001), whereas viable female offspring did not differ significantly between treatments (Figure 2C; *P =* 0.14). Sex ratio (SR-male/female) of F1s was not significantly different from a 1:1 in the single mating treatment (SR = 0.92; *P =* 0.71, Additional file 1: Supporting Information 1), but was male-biased in the multiple mating treatment (SR = 1.44; *P* = 0.03, Additional file 1: Supporting Information 1).

### F1 to F2

**Figure 3:**
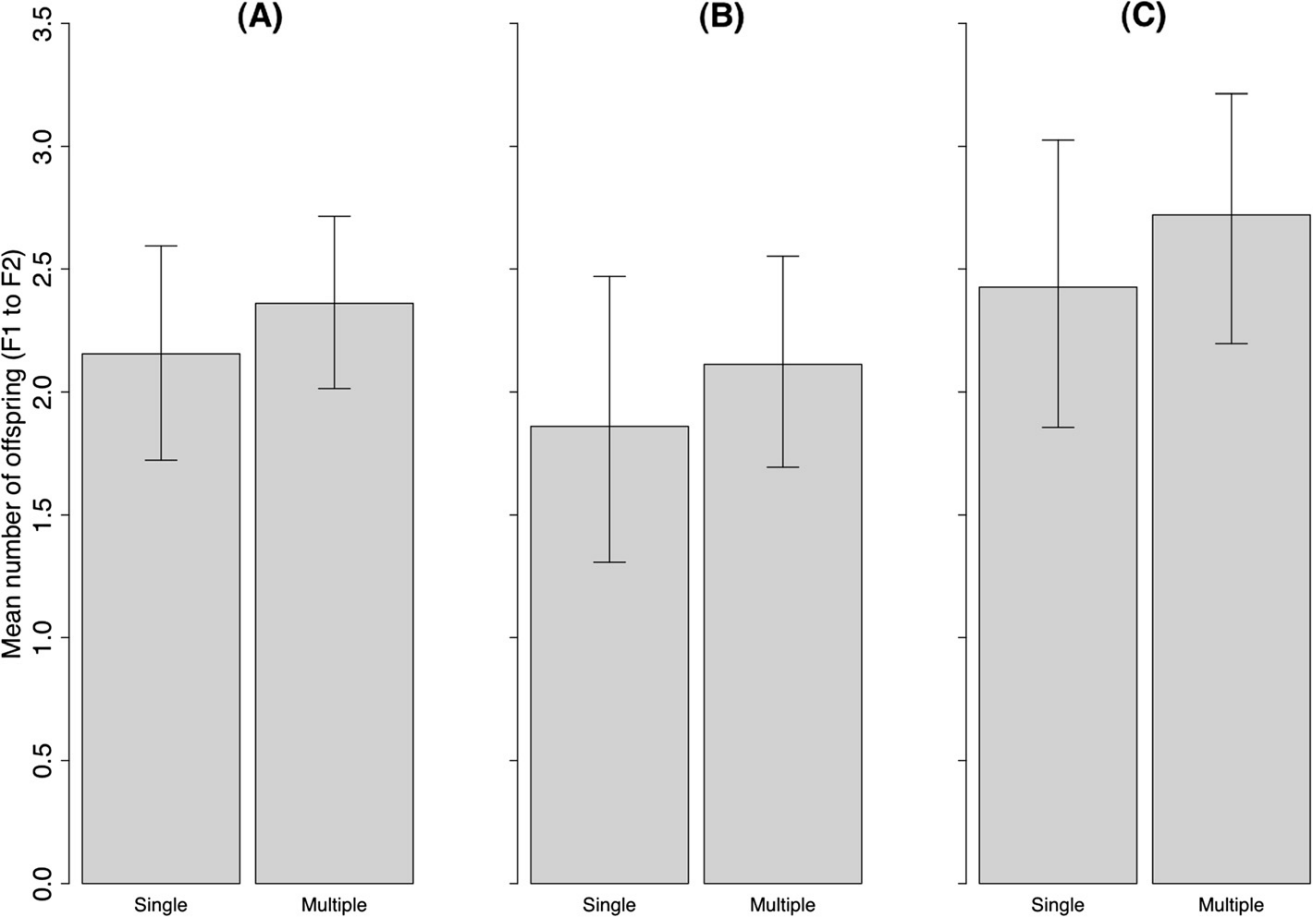
**Partitioning Fitness (F1 to F2).** Mean number of viable offspring (F2) produced by **(A)** all F1, **(B)** only male F1, and **(C)** only female F1. Whiskers indicate 95% bootstrap confidence intervals. Sample sizes used to calculate the means are shown in Supporting Information 1.

In the next generation, there were no significant differences in the reproductive success of individual F1 that had been produced from multiple versus single matings (Figure 3A; *P =* 0.78), regardless of whether the F1s were male (Figure 3B; *P =* 0.84) or female (Figure 3C, *P =* 0.67). In terms of sex ratios in the grand-offspring (F2), both treatments had even sex ratios that did not differ significantly from 1:1 (F2_Fo singly_; SR = 0.94, *P* = 0.61; F2_F0 multiply_; SR = 0.93, *P* = 0.40, Additional file 1: Supporting Information 1).

### Effect of multiple mating on size at birth, growth rate, time to sexual maturation and survival

Model selection revealed that for all traits, the estimated best model included a random effect due to tank of origin (i.e., random variation among F0 females), but no consistent difference between females in different treatments. There was also some support for a mixed effect model, which included treatment and tank (Table 2, Additional file 2: Supporting Information 2). Finally, in terms of survival, 96.7% of F1s from singly mated and 98% of F1s from multiply mated females survived up to 12 weeks. For F2s, 98% and 97.6% of grand-offspring, of singly and multiply mated origins respectively, survived until 12 weeks (Table 1, Additional file 1: Supporting Information 1). There was therefore no significant effect of multiple mating on F1 and F2 survival.

**Table 2:** Results for Mixed Effect models on Fitness components

**Size at birth**				
**F1**	Estimate	Std. error	DF	*P*
Fixed effect				
Intercept	−0.160	0.008	191	<0.001
Random effects	Std. Dev.			
Tank (Intercept)	0.068			
Residual	0.044			
**F2**	Estimate	Std. error	DF	*P*
Fixed effect				
Intercept	0.859	0.006	262	<0.001
Random effects	Std. Dev.			
Tank (Intercept)	0.020			
Residual	0.057			
Growth rate				
**F1**	Estimate	Std. error	DF	*P*
Fixed effect				
Intercept	−2.192	0.024	190	<0.001
Random effects	Std. Dev.			
Tank (Intercept)	0.184			
Residual	0.162			
**F2**	Estimate	Std. error	DF	*P*
Fixed effect				
Intercept	−2.120	0.041	274	<0.001
Random effects	Std. Dev.			
Tank (Intercept)	0.204			
Residual	0.190			
Time to sexual maturation				
**F1**				
Fixed effect	Estimate	Std. error	DF	*P*
Intercept	3.825	0.034	84	<0.001
Random effects	Std. Dev.			
Tank (Intercept)	0.253			
Residual	0.130			
**F2**				
Fixed effect	Estimate	Std. error	DF	*P*
Intercept	3.720	0.027	242	<0.001
Random effects	Std. Dev.			
Tank (Intercept)	0.174			
Residual	0.201			

Effect of mating treatment on size at birth, growth rate and time to sexual maturation in F1s and F2s using linear mixed-effects models. Tank of origin was used as a random effect nested within mating treatment. Parameters are only shown for best-fitted model. Selection of best-fitted model and Akaike weights are shown in Additional file 2: Supporting Information 2.

## Discussion

Despite growing evidence that females obtain reproductive benefits from mating multiply, the extent to which these benefits are partitioned between first- and second-generations remains controversial [6,7,13,39]. This partitioning is critical to validate the assumption that multiple mating confers genetic benefits that increase offspring net fitness (i.e., number of descendants produced). By comparing fitness gains, using multiple components of fitness, between singly and multiply mated females over two generations, we have addressed this question.

Our results strongly support the hypothesis that multiple mating is adaptive, as manifested in an increase in female fecundity. We found that multiply mated females produce substantially more grand-offspring than singly mated females. However, because the reproductive output (F2) of progeny from multiply and singly mated females was not significantly different, we also showed that this fitness advantage is driven by the production of more offspring in the first generation (F1), rather than by elevating the fitness of offspring (second-generation effects).

Our results do not preclude the possibility that selection for indirect benefits exists, because direct and indirect benefits may operate simultaneously [40]. Previous work has found the offspring of multiply mated female to be larger at birth, phenotypically more diverse, and have enhanced schooling and predator avoidance skills [17,41,42]. Such traits are likely to be important in the natural environment in enabling progeny to survive until they are able to reproduce. For example, there is evidence that environmental factors such as disease play a role in female indirect selection for males with pathogen resistance alleles [43]. A similar process could operate in naturally occurring guppy populations. Thus, while indirect benefits may operate under different environmental circumstances that were not investigated here, our study provides clear evidence that multiple mating benefits females, and that the direct effect of an increase in fecundity plays a key role in delivering this benefit. Importantly, mating treatment had no effect in terms of terms of size at birth, growth rate or viability, underscoring the importance of the scale of fecundity fitness benefits from multiple mating.

Fitness can be defined as a measure of the proportion of individuals that are propagated into the following generations [26]. A limitation of our study was that net fitness (i.e., number of descendants produced) was only recorded for the first brood. Nevertheless, we do not believe that this materially undermines our conclusions. Specifically, in contrast to individuals exposed to natural variation (in which fitness fluctuates according to the different environments experienced), all the individuals of F0, F1, and F2 generations in our experiment were allocated to identical conditions of light, temperature, food provisioning, and sexual harassment. Under such stable and identical conditions, temporal variation in fitness is likely to be small [30]. Also, by recording fitness on a large number of tanks per mating treatment across two generations, we reduced the risk of getting a biased estimate of fitness [27,28].

It is possible that singly mated F0 females produced fewer F1s as result of brood retention, but we think this possibility is unlikely. One advantage of multiple mating over single mating is that it enables mechanisms of post-copulatory sexual selection to operate to maximize fitness [3]. If F0 females in the singly mated treatment retained brood production, then this can be seen as an advantage of multiple mating over single mating. It is, however, important to point out that there were no differences in the probability of producing a first brood or in the time of gestation between singly and multiply mated F0 females (see results). These two variables are good indicators of female stress. Additionally, guppies lack superfetation, meaning that all embryos are at the same developmental stage [44]. It is therefore unlikely that brood retention could explain our results.

When the environment is controlled, as it was in this experiment, it is the increased quantity of F1 offspring, rather than the quality of those offspring, that is the key determinant of fitness. Previous work found the offspring of multiply mated female to be larger at birth, and phenotypically more diverse, and to have enhanced schooling, and predator avoidance skills [17,41,42]. Size at birth and growth rate are strongly maternally influenced [45], and have been shown to be important fitness correlates in guppies [46-48]. Although phenotypic diversity and offspring behavior were not measured, we found no effect of multiple mating on size at birth, growth rate, time to sexual maturity, or survival. In this, our results are consistent with a recent meta-analysis, which showed that female multiple mating does not have a significant impact on such offspring demographic traits [39]. The identical conditions across mating treatments and generations could have minimized selection for the traits analyzed in our study.

Similar increases in fecundity associated with multiple mating have been reported across different taxa [1,19,49,50]. Theoretical models predict that if direct benefits outweigh the costs associated with multiple mating, there will be enough selective pressure to maintain multiple mating based on direct reproductive benefits without the need for second generation fitness benefits [11,51]. In species with internal copulation, a major survival cost associated with multiple mating is the physical injury and disease transmission caused by the male’s sexual organ. Since the frequency of multiple mating is similar in laboratory and wild conditions [52], the above costs are therefore predicted to be comparable (although the prevalence of disease is expected to be greater in the wild). Multiple mating had no effect on survival or on the probability of producing a first brood in F0 females. Mating treatment also did not affect the number of F1s that reach sexual maturity, hence it is unlikely to have caused any difference in fitness. Although we cannot rule out the effect of “ideal laboratory conditions” in alleviating the costs associated with multiple mating, the fact that we did not find any effect of multiple mating in costs that are measurable under laboratory conditions and in any of the life history traits studied indicates the costs were negligible in the multiple mating treatment.

The greater fecundity in the multiple mating treatment was driven by the over-production of viable sons (> 87%). Likewise, multiply mated females of house wren produce a surplus of male offspring [53]. While theoretical models predict that female multiple mating can affect the dynamics of sex ratio [54], empirical studies documenting such effects are rare [55]. Here, we empirically show for the first time, that female multiple mating influences the offspring sex-ratio in the Trinidadian guppy. Sex ratios are usually female biased in laboratory guppy strains [56], which is in stark contrast to our results. Variation in environmental/social conditions affects sex ratios [46,57]. As water temperature, feeding and level of sexual harassment were identical between mating treatments, so a sex ratio adjustment in response to differences in environmental conditions seems unlikely. Evolutionary theory predicts that when conditions are good, females should invest more in the sex with greater reproductive variability [58], which in our case is expected to be the male sex. Multiple mating and female harassment by males is the norm among guppy populations, whereas single mating is the unnatural condition. Is it possible that this created stress that led singly mated females to produce fewer sons? Future studies should investigate the causes of this over production of sons from multiply mated females and ask whether this is adaptive in the long run.

One possible mechanism for the over-production of sons is the existence of segregation distortion genes or sex ratio meiotic drive. During spermatogenesis, the sex ratio distortion gene links to one of the sex chromosomes and prevents the production of functional gametes bearing the other sex chromosomes [59]. Sex ratio segregation distortion genes have been reported for many fish species, including guppies [60]. However, these genes act during spermatogenesis, which occurred before the start of the experiment, as it typically takes 36 days in guppies [61]. Hence it is unclear how segregation distortion genes could have differentially affected the two treatments unless they influence the outcome of sperm competition. In fact, in fruit flies, sperm bearing sex ratio distorting genes have reduced competitive ability, giving them a reduced share of paternity under contexts of sperm competition, such as those created by multiple mating [55,62].

## Conclusions

For most of the twentieth century, studies of sexual selection assumed that female fitness can be maximized by mating with a single male [63]. As a result, multiple mating by females was mostly seen as a consequence of sexual conflict and sexual coercion by males [38]. More recently there has been a shift in this perspective, with the female’s role in multiple mating increasingly recognized [64-66]. Our study reinforces this idea and adds to the growing evidence that the benefits gained by females from multiple mating in most cases outweigh the costs. As our study shows, under benign conditions, multiple mating brings a ~1.5 fold increase in female fecundity. This increase in fecundity, which occurs at an apparently negligible physical cost for both the mother and offspring, is a strong indicator of the adaptiveness of female multiple mating. Sexual conflict arises when the reproductive agendas of each sex are different [67]. However if multiple mating is advantageous for females, as is increasingly recognized across many taxa, then it is time to examine mating decisions from the perspective that both female and male fitness can be maximized by mating multiply. This does not mean that sexual conflict is irrelevant, but it shows that the effects of female multiple mating on fitness must also always be considered.

## Methods

### Experimental design

We used descendants of wild caught guppies from the Lower Tacarigua River, Trinidad, to generate virgin females and males that were later used to generate singly and multiply mating broods. Sixty pregnant females were haphazardly selected and transferred to single 10 L tanks and allowed to give birth. Of the 60 females, 51 produced broods that provided the first generation of fish (F0) used in our experiment. After birth each offspring was allocated to a single 10 L tank for 12 weeks at which point sex could be unambiguously determined. All individuals were kept in identical laboratory conditions. Tanks were filled with de-chlorinated tap water, contained clean natural gravel and maintained at approximately 20–24°C under a 12-hour light/dark regime. Each tank had its own filter. All individuals were fed ad libitum daily with live artemia.

At three months old, F0 females and F0 males were haphazardly allocated to either a single or a multiple mating treatment. In both mating treatments, each female had access to only one male at any given time. After the first day, all males were removed and allocated to individual tanks for 24 hours. On the second day, in the single mating treatment the same male was introduced to the same female as in day 1, whereas in the multiple mating treatment a novel male was introduced to the female. This process was repeated for the next two days, with the same male introduced to the singly mated female and a new male introduced to the multiply mated female. F0 males allocated to the multiple mating treatment were not rotated among different replicates (i.e., each group of four males was only used in one replicate). In both mating treatments, F0 females were allowed to settle for 24 hours before mating trials began. F0 males were introduced the following day at 0700 and removed at 1700. We adopted a similar experimental design to that used by Tregenza and Wedell (1998) [68], in which the mating frequency remained constant between mating treatments whereas the number of mates varied. This mimics the chance encounters with males that occur under natural conditions, while controlling for potential confounding effects linked to sexual harassment, which are known to strongly influence mating in this species [69]. At the end of the fourth day, all F0 males were removed, and F0 females were kept individually in their home tanks until either a first brood was produced or 90 days had elapsed.

All tanks were inspected for newborns twice a day (morning and afternoon). After the birth of the first brood the F0 female was allocated to a stock tank and not used again. F0 females that failed to successfully produce a brood were removed and replaced by a new female. Therefore, to obtain a total of 40 broods for each treatment, 73 and 78 F0 females were mated for the single and multiple mating treatment, respectively (Table 1, Additional file 1: Supporting information 1). These numbers of unsuccessful attempts were used to test for differences in the probability of mating success (see below). Immediately after birth the standard length of every individual F1 offspring was measured (Table 1). All 121 and 154 offspring of singly and multiply mated females (respectively) were transferred to individual and separated 10 L tanks and kept there for 12 weeks. Growth rate was calculated by recording the standard length of each individual F1 every week for 12 weeks (Table 1). Time to sexual maturation was also recorded for male F1s (Table 1). A male was considered sexual mature when the gonopodium extended beyond the tip of the anal fin.

After 12 weeks, each F1 offspring was presented with either a virgin female or male (these individuals were reared in individual tanks and used only as pairs for F1s), according to its sex, of similar size, and allowed to mate freely until a first F2 brood was produced. In contrast with the F0s, F1s were allowed to mate indeterminately until either a first brood of F2s was produced, or one of the F1 fish died. After the birth of the first F2 brood, a random sample of F2s had their size at birth, growth rate and time to sexual maturation recorded (Table 1). Size at birth, growth rate, and time to sexual maturation was only recorded for a sub set of F2s because of space limitations in the laboratory that prevented us from allocating all F2s to individual tanks. Finally, survival rate was recorded for F1s and F2s by recording whether a given individual survived until the end of the study (12 weeks) (Table 1).

All behavioural observations were carried out at the School of Biology at the University of St Andrews. The premises where the observations were carried out comply with the ASAB Guidelines for the treatment of animal in behavioural Research and Teaching, set by UK Home Office (PCD 60/2609).

### Statistical design

We first tested whether multiply mated females had a significantly higher probability of producing a first brood than singly mated ones (i.e., breeding success). Because the response variable for calculating breeding success is dichotomous (i.e., a females either produces a brood, or not), and matings were conducted until 40 replicates per mating treatment were obtained, the appropriate probability distribution is the negative binomial (i.e., the probability distribution for the number of “trials” required to obtain a pre-determined number of “successes” [70]). Consequently, the appropriate log-likelihood is:

(1) $$\begin{array}{ll} \log\left(L\right)= & \log\left(\frac{\left(N_{s}-1\right)!}{\left(k_{s}-1\right)!\left(N_{s}-k_{s}\right)!}\right)-k_{s}\log\left(p_{s}\right)\\ & +\left(N_{s}-k_{s}\right)\log\left(1-p_{s}\right)\\ & +\log\left(\frac{\left(N_{m}-1\right)!}{\left(k_{m}-1\right)!\left(N_{m}-k_{m}\right)!}\right)\\ & -k_{m}\log\left(p_{m}\right)+\left(N_{m}-k_{m}\right)\log\left(1-p_{m}\right) \end{array}$$

where the subscripts *s* and *m* refer to the singly and multiply mated treatments, respectively, *N*_*i*_ is the number of “trials” (i.e., number of F0 females for whom mating was attempted) in treatment *i* (*i = s* or *m*), *k*_*i*_ is the pre-specified number of “successes” (F0 females that produced a brood: 40 for each treatment, in our case) in that treatment, and *p*_*i*_ is the probability that a randomly chosen F0 female will successfully produce a brood in that treatment. *N*_*i*_ and *k*_*i*_ are the data, and the *p*_*i*_ are the parameters that must be estimated. To test for differences in breeding success between mating treatments, we fitted two versions of the likelihood in eq. (1): one in which breeding success differed between mating treatments (i.e., *p*_s_ and *p*_m_ were estimated as distinct parameters (*p*_s_ ≠ *p*_*m*_), and a second in which the multiply and singly mated females had the same probability of success (*p*_*s*_*= p*_*m*_*= p*). We wished to test the null hypothesis of equal mating success. We therefore fitted the mating success data to the negative binomial distribution under our null hypothesis (*p*_*s*_*= p*_*m*_*= p*), and our alternative hypothesis (*p*_*s*_ ≠ *p*_*m*_), and we determined whether the null hypothesis of equal mating success could be rejected with 95% confidence by comparing the two models with a likelihood ratio test.

Second, we tested whether the average number of viable grand-offspring produced (i.e., those surviving to adulthood; ~12 weeks) was greater for multiply mated females than for singly mated ones. No standard parametric distribution provided a satisfactory characterization of the number of grand-offspring per successful brood. Therefore, we used non-parametric bootstrapping, which makes no distributional assumptions about the data [71], to characterize the uncertainty around the number of grand-offspring per successful F0 brood. To test whether the average number of viable grand-offspring (i.e., those surviving to adulthood [~12 weeks]) produced was greater for multiply mated females than for singly mated ones, we used a combination of parametric (for breeding success) and nonparametric (for number of grand-offspring per successful F0 brood) bootstrapping. First, for *p*_*s*_ and *p*_*m*_, we randomly drew a probability of breeding success from the uncertainty distribution around our negative binomial maximum likelihood estimate (MLE) of this quantity (which we obtained from the inverse of the second partial derivative of the likelihood function, according to standard likelihood theory). Second, we used non-parametric bootstrapping to produce an uncertainty distribution for the mean number of viable grand-offspring produced per F0 female (i.e., number of F1 that survive 12 weeks). By randomly drawing a value of *p*, and a value for the mean number of viable grand-offspring from their respective uncertainty distributions, and then multiplying them together, we obtained a bootstrap replicate of the mean number of offspring produced per mating. By following the same bootstrap procedure for the singly mated female, and then subtracting the latter from the former, we obtained a bootstrap replicate of the difference in average number of offspring produced per mating treatment. We repeated this process 1000 times, and judged multiple mating as significantly more successful if >95% of the bootstrapped differences were greater than zero.

In addition, to gain more insight into the proximate mechanisms by which any differences in reproductive success arose, we conducted two further sets of analyses. We used a bootstrap analysis similar to that described above (except that viable sons and daughters were counted, rather than grand-offspring) to estimate the difference in average number of viable F1 offspring produced from multiple versus single matings. We also estimated the difference in the average number of F2 produced between F1 offspring of multiply mated mothers and F1 offspring of singly mated mothers. In F1s, however, a fixed number of individuals were mated (rather than mating occurring until a fixed number of successes occurred), so the appropriate likelihood for estimating the probability of breeding success for F1 individuals was the binomial distribution, rather than the negative binomial distribution.

(2) $$\begin{array}{ll} \log\left(L\right)= & \log\left(\frac{N_{s}!}{k_{s}!\left(N_{s}-k_{s}\right)!}\right)-k_{s}\log\left(p_{s}\right)\\ & +\left(N_{s}-k_{s}\right)\log\left(1-p_{s}\right)\\ & +\log\left(\frac{N_{m}!}{k_{m}!\left(N_{m}-k_{m}\right)!}\right)-k_{m}\log\left(p_{m}\right)\\ & +\left(N_{m}-k_{m}\right)\log\left(1-p_{m}\right) \end{array}$$

Additionally, we also compared sizes at birth, growth rates and time to male sexual maturation between mating treatments, to determine whether any differences in brood size were being traded off against any other fitness related traits. Both of these response variables followed an approximately Gaussian distribution after log-transformation, allowing application of a more conventional statistical analysis. In the analysis of brood size, there is only one response variable value per parent (number of progeny). For birth, growth, and time to sexual maturation, however, we have replication within parents (i.e., each offspring contributes a value). Because parental effects on these traits are likely, we treated each parent as a random effect nested within mating treatment, and fitted the data with linear mixed-effects models (function glmmPQL in R) [72]. For model selection, we used Akaike’s Information Criterion [73]. Specifically, we calculated AIC, the difference between the AIC of each model, and that of the estimated best model (the model with the lowest AIC). We also calculated Akaike weights, which are estimates of the probability that each model is actually the best in the model set. Thus, if multiple models have similar Akaike weights, then there is some uncertainty about which model is best.

For all analyses involving brood size, reported *P-*values were obtained from percentiles of the bootstrap distributions generated by the bootstrap analyses described above. For the analyses of size, growth and time to maturation, *P-*values were obtained from the fitted generalized linear mixed effects models. For the analysis of brood success, *P-*values were obtained from the likelihood ratio test (i.e., the chi-squared distribution with one degree of freedom). All analyses were performed using R 2.14.0 [74].

## Competing interests

The authors declare that they have no competing interests.

## Authors’ contributions

MB, AEM conceived the idea and designed the experiment. MB carried out the experiment. MB, SRC, MH, MD, performed the statistical analyses. MB, SRC, MH, MD, AEM, interpreted the results and wrote the manuscript. All authors read and approved the final manuscript.

## Supplementary Material

Additional file 1 **Supporting Information 1.** Total number of individuals and the descendants produced, which were used to calculate the means and confidence intervals for Figures 1, 2 and 3. Viable individuals are those that reach sexual maturation and breeding success is the number of those that successfully produce a first brood. Click here for file

Additional file 2 **Supporting Information 2.** Model selection using values of ΔAIC (Akaike weights). k: Number of parameters of the model. The estimated best fitting model is shaded in grey. Click here for file
