# Supplementary material for: Fitness consequences of female multiple mating: A direct test of indirect benefits
Source: BMC Evol Biol. 2012 Sep 15;12:185. doi: 10.1186/1471-2148-12-185 (PMC3499236; doi:10.1186/1471-2148-12-185)
Supplement: Additional file 1 — Supporting Information 1. Total number of individuals and the descendants produced, which were used to calculate the means and confidence intervals for Figures 1, 2 and 3. Viable individuals are those that reach sexual maturation and breeding success is the number of those that successfully produce a first brood. [file 1471-2148-12-185-S1.pdf]

Supporting Information 1 – Total number of individuals and the descendants produced, which were used to calculate the means and confidence intervals for Figures 1, 2 and 3. Viable individuals are those that reach sexual maturation and breeding success is the number of those that successfully produce a first brood.

	Total (N)	Viable	Breeding success
F0 single	73	40	40
F0 multiple	58	39	39
F1 *via* single F0	121	119	85
F1 *via* multiple F0	154	147	115
F1 daughter *via* single F0	63	61	46
F1 son *via* single F0	58	58	39
F1 daughter *via* multiple F0	63	60	50
F1 son *via* multiple F0	91	87	65
F2 *via* single F0	335	324	
F2 *via* multiple F0	436	418	
F2 granddaughters *via* single F0	335	167	
F2 grandsons *via* single F0		157	
F2 granddaughters *via* multiple F0	436	217	
F2 grandsons *via* multiple F0		201	
F2s *via* F1 daughters *via* single F0	197	191	
F2s *via* F1 sons *via* single F0	140	133	
F2s *via* F1 daughters *via* multiple F0	200	192	
F2s *via* F1 sons *via* multiple F0	236	226	
