# Supplementary material for: Fitness consequences of female multiple mating: A direct test of indirect benefits
Source: BMC Evol Biol. 2012 Sep 15;12:185. doi: 10.1186/1471-2148-12-185 (PMC3499236; doi:10.1186/1471-2148-12-185)
Supplement: Additional file 2 — Supporting Information 2. Model selection using values of ΔAIC (Akaike weights). k: Number of parameters of the model. The estimated best fitting model is shaded in grey. [file 1471-2148-12-185-S2.pdf]

Supporting Information 2 – Model selection using values of ΔAIC (Akaike weights). k: Number of parameters of the model. The estimated best fitting model is shaded in grey.

		Size at birth		Growth rate		Time to sexual maturation	
Model	k	F1	F2	F1	F2	F1	F2
Single-mean	1	185.9	88.1	124.9	191.1	92.80	75.27
		(<0.001)	(<0.001)	(<0.001)	(<0.001)	(<0.001)	(<0.001)
Fixed-effect (treatment)	2	187.0	88.9	121.0	192.1	94.00	71.64
		(<0.001)	(<0.001)	(<0.001)	(<0.001)	(<0.001)	(<0.001)
Random-effect (tank)	3	0.0	0.0	0.0	0.0	0.0	0.0
		(0.680)	(0.723)	(0.639)	(0.731)	(0.731)	(0.580)
Mixed-effect (treatment, tank)	4	1.5	1.9	1.1	2.0	2.0	0.633
		(0.320)	(0.277)	(0.361)	(0.269)	(0.269)	(0.421)
